# Can Tweets Predict Citations? Metrics of Social Impact Based on Twitter and Correlation with Traditional Metrics of Scientific Impact

**DOI:** 10.2196/jmir.2012

**Published:** 2011-12-16

**Authors:** Gunther Eysenbach

**Affiliations:** ^1^University Health NetworkCentre for Global eHealth Innovation & Techna InstituteToronto, ONCanada; ^2^Institute for Health Policy, Management, and EvaluationUniversity of TorontoToronto, ONCanada; ^3^JMIR Publications Inc.Toronto, ONCanada

**Keywords:** bibliometrics, blogging, periodicals as topic, peer-review, publishing, social media analytics, scientometrics, infodemiology, infometrics, reproducibility of results, medicine 2.0, power law, Twitter

## Abstract

**Background:**

Citations in peer-reviewed articles and the impact factor are generally accepted measures of scientific impact. Web 2.0 tools such as Twitter, blogs or social bookmarking tools provide the possibility to construct innovative article-level or journal-level metrics to gauge impact and influence. However, the relationship of the these new metrics to traditional metrics such as citations is not known.

**Objective:**

(1) To explore the feasibility of measuring social impact of and public attention to scholarly articles by analyzing buzz in social media, (2) to explore the dynamics, content, and timing of tweets relative to the publication of a scholarly article, and (3) to explore whether these metrics are sensitive and specific enough to predict highly cited articles.

**Methods:**

Between July 2008 and November 2011, all tweets containing links to articles in the Journal of Medical Internet Research (JMIR) were mined. For a subset of 1573 tweets about 55 articles published between issues 3/2009 and 2/2010, different metrics of social media impact were calculated and compared against subsequent citation data from Scopus and Google Scholar 17 to 29 months later. A heuristic to predict the top-cited articles in each issue through tweet metrics was validated.

**Results:**

A total of 4208 tweets cited 286 distinct JMIR articles. The distribution of tweets over the first 30 days after article publication followed a power law (Zipf, Bradford, or Pareto distribution), with most tweets sent on the day when an article was published (1458/3318, 43.94% of all tweets in a 60-day period) or on the following day (528/3318, 15.9%), followed by a rapid decay. The Pearson correlations between tweetations and citations were moderate and statistically significant, with correlation coefficients ranging from .42 to .72 for the log-transformed Google Scholar citations, but were less clear for Scopus citations and rank correlations. A linear multivariate model with time and tweets as significant predictors (P < .001) could explain 27% of the variation of citations. Highly tweeted articles were 11 times more likely to be highly cited than less-tweeted articles (9/12 or 75% of highly tweeted article were highly cited, while only 3/43 or 7% of less-tweeted articles were highly cited; rate ratio 0.75/0.07 = 10.75, 95% confidence interval, 3.4–33.6). Top-cited articles can be predicted from top-tweeted articles with 93% specificity and 75% sensitivity.

**Conclusions:**

Tweets can predict highly cited articles within the first 3 days of article publication. Social media activity either increases citations or reflects the underlying qualities of the article that also predict citations, but the true use of these metrics is to measure the distinct concept of social impact. Social impact measures based on tweets are proposed to complement traditional citation metrics. The proposed twimpact factor may be a useful and timely metric to measure uptake of research findings and to filter research findings resonating with the public in real time.

## Introduction

Scientists, research organizations, and funding agencies require metrics to measure the impact of research. Citations in peer-reviewed articles referencing other articles are a widely accepted measure of scientific impact. Citations are the basis for metrics like the h-index [1] and its derivatives, which are used to evaluate the productivity and impact of individual researchers, or the impact factor, which is used to evaluate the scientific impact of journals [2]. However, citations as a metric have various disadvantages, including the fact that they take a very long time to accumulate. They are also difficult to obtain (in an environment where the majority of research is still not open access) and are often available only in proprietary databases; thus, these metrics are not necessarily transparent or reproducible. For example, the h-index of a researcher varies widely depending on the database used to calculate it, and calculation of the journal impact factor has been criticized for not being transparent [3,4]. Finally, citations measure only uptake within and impact on the scientific community, not, for example, impact on or dissemination among knowledge users (policy makers, patients, and the general public). While this may be desirable for some use cases, other applications and stakeholders require a broader definition of impact. Concepts such as impact on society, social impact, real-world impact, knowledge translation, and uptake by the public should be part of every research assessment exercise but are notoriously difficult to measure [5]. Tools such as questionnaires applied to publications have been suggested to measure the “societal impact factor” [6], but it is unclear whether these instruments, which require manual data collection, are scalable to a large number of publications.

In this paper I propose new metrics and a new source of data—Twitter—that could be used to measure social impact, complementing traditional citation analyses, pilot tested and illustrated on a set of articles from the *Journal of Medical Internet Research* (JMIR).

Web citation analysis has previously been used to measure the extent to which articles or ideas are mentioned on the Web [7]. For example, Vaughan and colleagues have shown relationships between link metrics [8] or Web mentionings [9,10] and traditional impact metrics. Kousha and colleagues propose an “integrated online impact indicator” [11], which combines a range of online sources into one indicator for impact on the Web, including course reading lists, Google blogs, PowerPoint presentations [12], and Google Books [13].

Web 2.0 tools such as Twitter and blogs, as well as social bookmarking tools and Web-based reference management tools such as CiteULike and Mendeley, provide the opportunity to gather novel metrics from other sources that provide data in a structured format, accessible through application programming interfaces (APIs) [14,15]. These metrics—sometimes called altmetrics [16] or (in a broader context) infodemiology metrics [17,18]—can be used to gauge concepts such as popularity, buzz, social impact, or uptake of new information. The underlying common idea is that scientists and the public leave digital traces on the Internet when searching for or using information, and the Web has “made measurable what was previously immeasurable,” [18] which is the demand for or use of specific information, and dissemination of information, as it propagates through networks. Infodemiology is an emerging area of science with applications in public health [17,18] and a wide range of other areas [19]—it has, for example, been shown that search engine queries predict influenza [20,21], that tweets during the H1N1 pandemic correlated with incidence rates [22], and that tweets about a movie accurately predict its box-office success before the movie is even released [23].

In analogy to the applications for public health 2.0 [17], economics, and other areas [19], there is an obvious application of infodemiology or infoveillance for *scientometrics 2.0* [24], which is to study the buzz around scientific publications to measure or even predict the impact of research.

The field of social media-based scientometrics (altmetrics, infodemiology metrics) is in its infancy, and many open questions need to be addressed. It may be that these new metrics measure completely different concepts that are not correlated with other traditional metrics such as citations, but it may also be that important publications in the scholarly literature first lead to a measurable buzz within the blogosphere (and other Web 2.0 venues) before, years later, the buzz is also reflected in increased citations and/or policy changes and social impact.

Specific questions include the following. (1) How can buzz be measured? (2) When (in relation to the publication of an article) and how long should we measure it? (3) If we can measure something, how are the metrics related to traditional metrics such as citations, and is the buzz sensitive enough to predict increased citations? (It should be noted that prediction of citations is not necessarily the end goal, and that lack of correlation is not necessarily a failure, because it is clear that these metrics add a new dimension of measuring impact.)

There is a dearth of empirical data exploring and showing such relationships, which would be seminal to develop the field of social media-based scientometrics. While it has been shown that scholars cite on Twitter and reasons for scholars to do so have been explored [24], little is known how—on an article- or journal-level—publications attract tweets, and whether meaningful metrics can be derived.

There is a small but quickly growing body of literature focusing on Twitter for use in scholarship [24-29]. Most papers focus on analyzing Twitter streams collected during conferences [25-27], while little or no evidence is available on a journal level. The Public Library of Science (PLoS) journals make available some article-level impact metrics, which scholars have started to analyze [30], but PLoS has only recently begun to count tweets.

At JMIR we started the current empirical, prospective study in 2008, at a time when few journal publishers or scholars thought about the potential of Twitter for analyzing impact. The goals of the current study were (1) to explore the content and characteristics of tweets discussing or mentioning research articles and their timing relative to the publication date of an article, (2) to identify suitable metrics to describe propagation of new evidence through social media networks, and (3) to explore how the proposed metrics correlate with traditional metrics of uptake within the scientific community (traditional citations).

## Methods

### JMIR Twitter Dataset and Tweetation Counts

**Figure 1:**
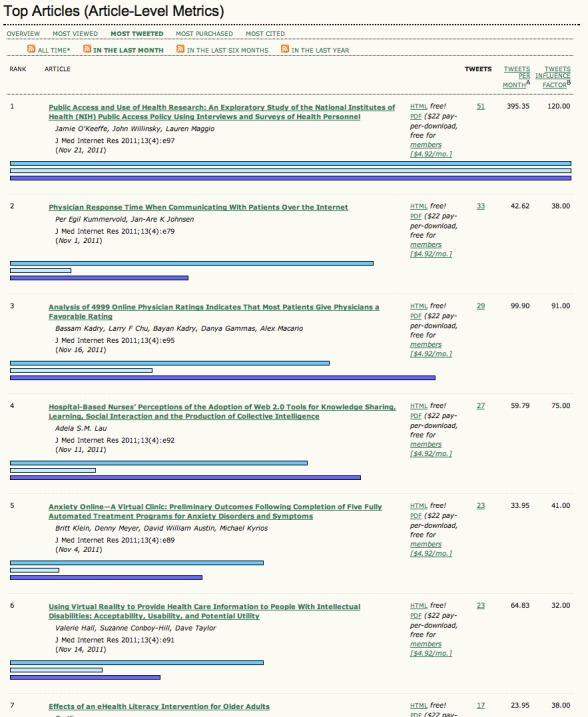
Top Articles ranking on the *Journal of Medical Internet Research* (JMIR) (sorted by most-tweeted articles in November 2011).

JMIR is a leading, highly cited open access journal with a Thomson Reuters (formerly ISI) 3-year impact factor of 4.7 and 5-year impact factor of 5.0 (Journal Citation Reports, 2010). In July 2008, it was the first journal to start systematically mining tweets that mention its published articles, showing them in real time on the JMIR “Top Articles” Page (see Figure 1). Data are collected using the Twitter Search API. 

For the purpose of this paper, I call a citation in a tweet (mentioning a journal article URL) a “tweetation”, to distinguish it from a citation in a journal article (which is the metric I compared tweetations against). As 1 tweet can have multiple tweetations (a tweet containing multiple different URLs citing different articles), the number of tweetations is not necessarily identical to the number of tweets, although in our sample a tweet with multiple tweetations was very rare, so that I sometimes use *tweets* and *tweetations* interchangeably. Only tweets with URLs linking directly to the journal article are captured—that is, links to newspaper articles mentioning published research in JMIR or links to JMIR articles that are not on the JMIR site (eg, instances in PubMed Central, or links to the digital object identifier [DOI] handle)—are not counted. Retweets of the same tweet or sending a modified tweet by other users would count as multiple tweetations, as would multiple tweets from the same user containing the same URL.

### Citation Counts

Citation counts were harvested from Scopus and Google Scholar. The current study is based on citation counts obtained in November 2011, which is 17–29 months after the cited papers were published.

### Analysis

For the tweets distribution analysis all tweets sent and archived by JMIR between July 24, 2008 and November 20, 2011 were included (Multimedia Appendix 1).

For the tweetation–citation correlation analysis, I included only tweets that referred to articles published in issue 3/2009 through issue 2/2010—that is, tweetations of all 55 articles published between July 22, 2009 and June 30, 2010 (Multimedia Appendix 2). I chose this period because the tweetation rate for earlier articles was too sparse, and later articles did not have enough citations accumulated as of November 2011. 

Pearson correlations on the raw and the log-transformed data, as well as the Spearman rank correlations, were calculated. Data were log transformed using the natural logarithm because tweetation and citation data are highly skewed. As the log of 0 is undefined, 1 was added to the counts of citations and tweetations.

For the categorical classification analysis (attempts to predict highly cited articles from highly tweeted articles), “highly cited” articles were defined as articles that were in the top 25^th^ percentile of each issue (articles ranked by citation counts), and “highly tweeted” articles were defined as articles that were in the top 25^th^ percentile of each issue (ranked by tweetations). 

The analysis was stratified on a quarterly per-issue basis to adjust for time as a confounder, because the popularity of Twitter (and the number of JMIR followers) increased over time (older articles will have fewer tweets than newer articles), and because older articles will have more citations than more recent ones. Stratification by journal issue assures that the articles that were compared against each other were all published within the same quarter (3-month window).

In another analysis I included articles from all issues, but adjusted for time as a potential confounder by conducting a linear regression analysis, with the logarithm of citations as dependent variable, and time (days since publication of the earliest article in our dataset) and the logarithm of tweetations as independent variables.

Note that when article IDs are mentioned in this paper (see figures), these are part of the DOI; and each article can be identified by entering http://dx.doi.org/10.2196/jmir.{articleID} in a Web browser’s address bar.

## Results

### Average Number of Tweets per Article

A total of 4208 tweetations were identified, which cited a total of 286 distinct JMIR articles, with each article receiving on average 14 tweetations (median 9). However, these averages should be interpreted with care, as JMIR has published articles since 1999 (560 articles in total). Among the 286 articles referenced in tweetations, there were many articles that were published before data collection began or before Twitter even existed. As these older articles receive only sporadic tweetations, the average and median are not reflective of more recent articles.

**Figure 2:**
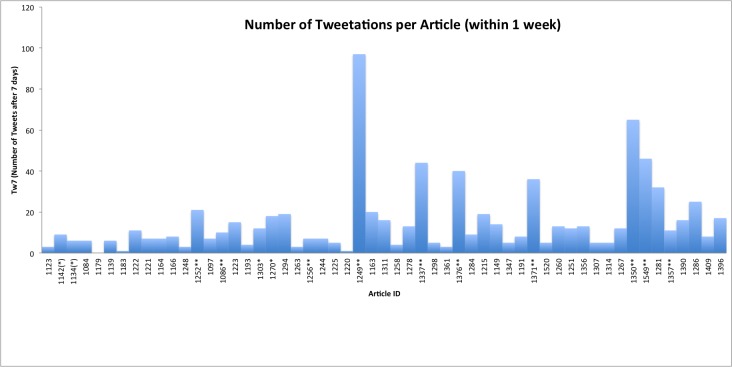
Number of tweetations within 7 days of article publication, per article ID. Asterisks next to article IDs denote that the article is top-cited (see also Figure 8): ** top 25th citation percentile within issue by both Scopus and Google Scholar citation counts * top 25th citation percentile according to Google Scholar only, (*) top 25th citation percentile according to Scopus only.

The 55 articles published in issues 3/2009–2/2010 received an average of 21.2 tweetations within 356 days after article publication (median 12, range 0–149), and 13.9 (median 8, range 0–96) tweetations within 7 days. Figure 2 shows the cumulative number of tweetations within 7 days (tw7) for these articles.

### Tweet Dynamics

**Figure 3:**
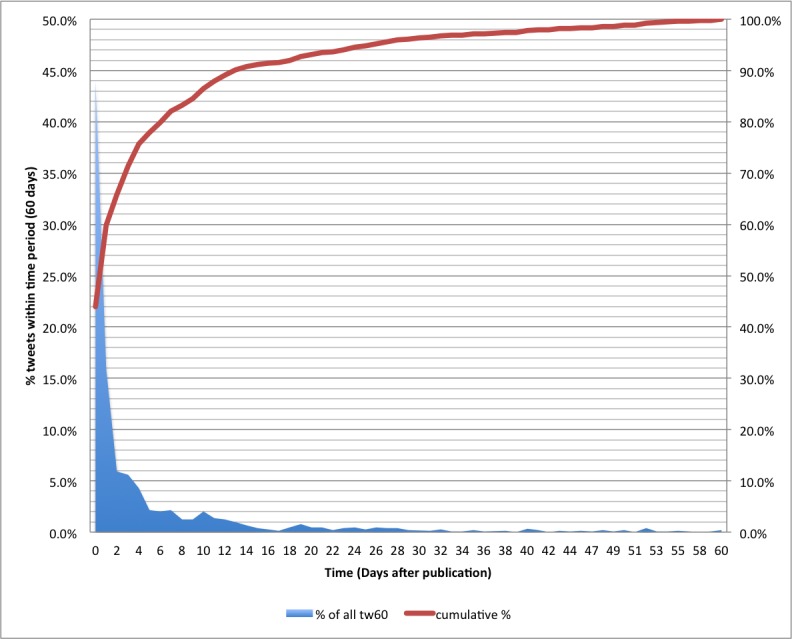
Tweetation dynamics. The blue, shaded area (left y-axis) shows the tweet rate (new tweetations per day, as a proportion of all tweetations during the first 60 days [tw60]). The red line (right y-axis) represents cumulative tweetations.

When, in relationship to the date of publication of an article, did the tweetations occur? Figure 3 shows the general distribution of all tweetations (n = 3318) that were sent within 60 days after publication of the article they are citing, by day. In this graph, day 0 refers to the day of article publication, day 1 is the following day, and so on; the left y-axis shows how many of the tweetations were sent on that day (tweet rate), as a proportion relative to all tweetations within a 60-day period; and the right y-axis (and red line) shows the cumulative proportion. The majority of tweets were sent on the day when an article was published (1458/3318, 43.9%) or on the following day (528/3318, 15.9%). Only 5.9% (197/3318) of all tweetations are sent on the second day after publication, and the downward trend continues, until a little plateau between days 5 and 7 occurs (about 2% of all 60-day tweetations). There is a dip on days 8 and 9, which may be explained by the fact that, while JMIR publishes articles on different days of the week, Friday is slightly more prevalent, so days 8 and 9 would fall on the following weekend. After day 10 (66/3318, 2%) the rate of new tweetations declines rapidly.

**Figure 4:**
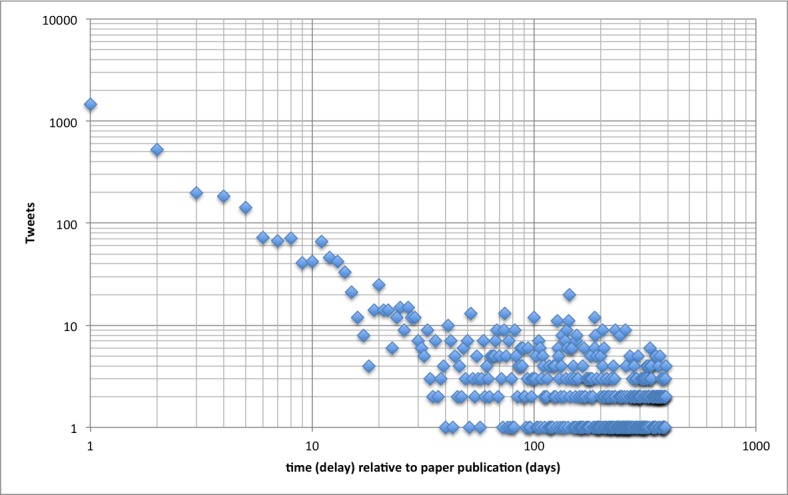
Tweetation dynamics over time on a log-log scale. All tweetations were categorized according to when, in relationship to the cited article publication date, they were tweeted (x-axis), with 1 being the day of article publication.

Figure 4 shows the same curve of new tweetations by day, but this time replotted with logarithmic horizontal and vertical axes. Now an interesting pattern emerges, showing a strong regularity: the tweetation distribution during the first 30 days on a log–log plot follows a straight line, which is indicative of a Pareto distribution, also known as Zipf’s law or Bradford distribution, which are said to follow a power law [31]. In our sample, the number of tweetations per day after the article has been published during the first 30 days can be predicted by the formula ln(tw) = –1.53 * ln(d) + 7.25, where tw is number of new tweetations on day d, and d is days since publication (publication date = day 1).

This model has an excellent fit (*R^2^* = .90). While the intercept of this formula is not important (it is dependent on the total number of tweetations), the term –1.53 is called alpha or the exponent of the power law (slope of the linear curve in the log–log diagram). 

We can divide the pattern in Figure 4 into two distinct phases: I call the first 30 days the “network propagation phase,” where the new information is propagated through the Twitter social network. After 30 days, the network propagation phase gives way to what I call the “sporadic tweetation phase,” where only sporadic mentionings of older articles and small clusters of localized outbreaks of information propagation occur.

**Figure 5:**
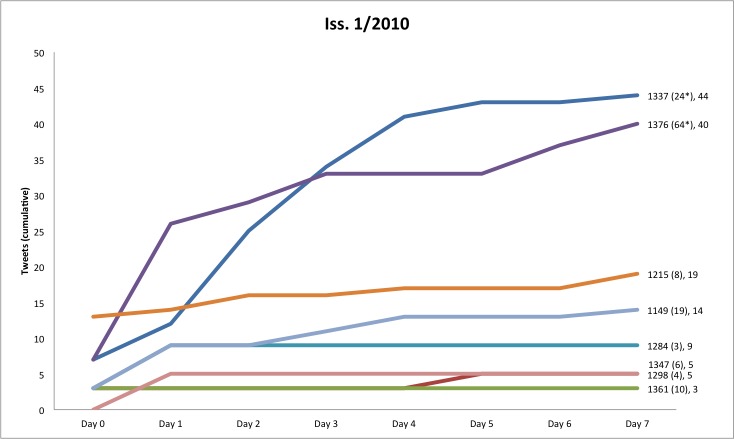
Tweetation dynamics in the first 7 days after article publication for one specific issue. The 4-digit number is the article identifier (last digits of the DOI), number in parentheses is the citation count (as per Google Scholar, November 2011), and the last number is the (cumulative) number of tweets on day 7 (tw7).

Figure 5 shows the tweetation dynamics for all articles in JMIR issue 1/2010. Note that while Figure 4 shows the number of *new* tweetations per day (tweet rate, which is sharply declining), Figure 5 shows them in a cumulative manner. The figure illustrates how some articles attract tweets only on the first day, while some other articles continue to attract tweetations and are more widely retweeted. Incidentally, these are often articles that turn out to be highly cited, as shown in more detail below.

### Other Regularities

**Figure 6:**
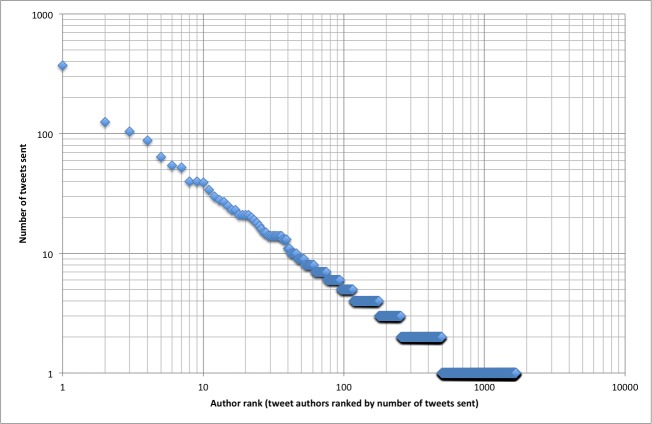
Tweetation density by account. Each Twitter account is ranked by the number of tweetations sent and plotted by rank on the x-axis. The y-axis shows how many tweetations were sent by each ranked account. For example, the top Twitter account ranked number 1 (@JMedInternetRes) sent 370 tweetations. Note the linear pattern on a log-log scale, implying a power law.

There were other strong regularities of tweetations following power laws. Tweetations were sent from 1668 distinct Twitter accounts (tweet authors). The most tweetations (n = 370) were sent by @JMedInternetRes, JMIR’s Twitter account. If we rank the accounts by the number of tweetations they sent and plot them against the number of tweetations for each account, the power law distribution shown in Figure 6 emerges. Half of all tweets (2105/4208, 50%) were sent by only 132 distinct tweet authors—that is, 8% of all tweet authors. The top 20% of the tweet authors (those ranked 1–334 by number of tweetations) accounted for 63.4% (2676/4208) of all tweetations. This uneven distribution of work is typical for Pareto distributions, an observation that is sometimes colloquially referred to as the 80/20 rule, where roughly 80% of the effects come from 20% of the causes.

**Figure 7:**
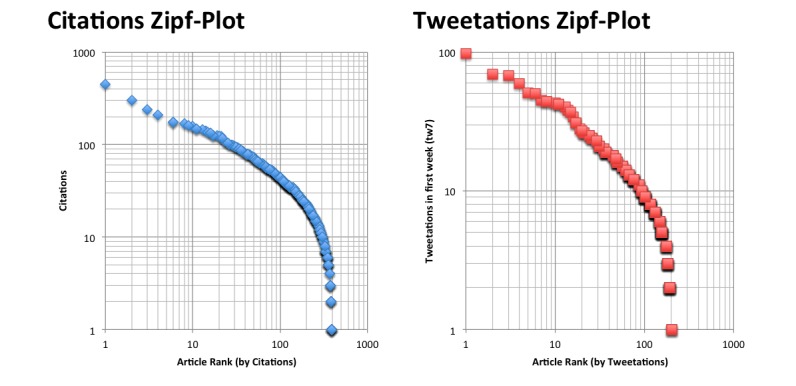
Left: Zipf plot for JMIR articles 3/2000-12/2009 (n=405), with number of citations (y-axis) plotted against the ranked articles. Right: Zipf plot showing the number of tweetations in the first week (tw7) to all JMIR articles (n=206) published between April 3, 2009 and November 15, 2011 (y-axis) plotted against the ranked articles. For example, the top tweeted article got 97 tweetations, the 10th article got 43 tweetations, and the 102th ranked article got 9 tweetations.

The third power law I looked at was where I expected it most, because this distribution is typically observed for citations and can be demonstrated in a Zipf plot, in which the number of citations of the *n*th most-cited paper is plotted versus the rank n (Figure 7, left). Tweetations follow a strikingly similar distribution (Figure 7, right).

### Citations

**Figure 8:**
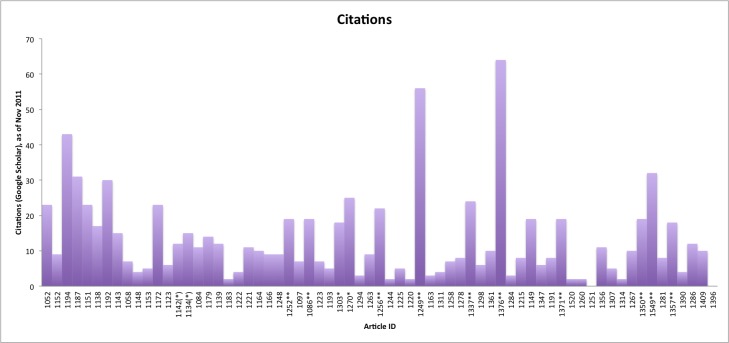
Google Scholar citation counts for all articles published between issue 3/2009 and issue 2/2010. Top-cited articles (75th percentile) within each issue are marked ** (top cited according to Google Scholar and Scopus), * (Google Scholar only), or (*) (Scopus only).

The 55 articles in our tweetations-versus-citations subset had an average of 7 citations on Scopus (median 4) and 13 citations on Google Scholar (median 9). Figure 8 shows the Google Scholar citation counts for all 55 articles included in the tweetation/citation analysis, as of November 2011.

First, the number of citations from Scopus were correlated with the number of citations from Google Scholar to test agreement between the two database sources. There was good agreement, with a Pearson correlation coefficient of .87 (*P* < .001) for the 55 articles. As Google Scholars’ citation counts were higher and appeared more robust, most results presented here refer to Google Scholar citation counts, unless noted otherwise.

**Figure 9:**
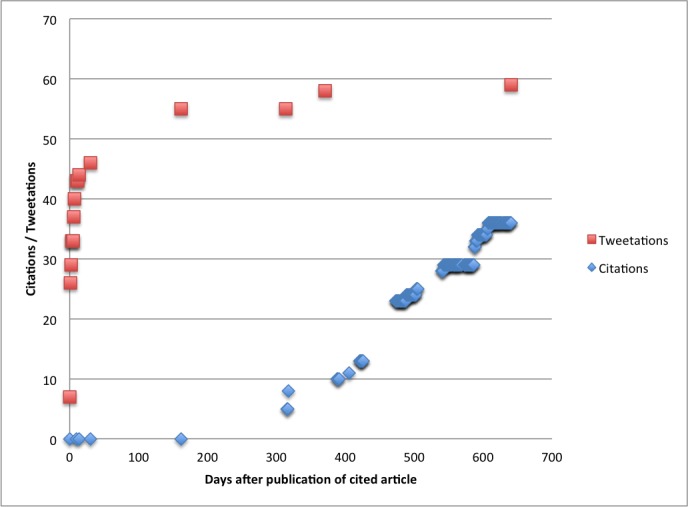
Citation and tweetation dynamics of a highly cited (and highly tweeted) article [article ID 1376]; citations according to Scopus.

Figure 9 compares a typical citation and a tweetation curve, illustrating the very different dynamics in tweetations compared with citations in scholarly articles. While citations in scholarly articles begin to accumulate only about 1 year after the article is published, tweetations accumulate mainly within the first few days after publication.

### Correlation Between Tweetations and Citations

For each journal issue, I separately plotted scatterplots and calculated Pearson correlation coefficients of the raw count, the logs, and Spearman rank correlation coefficients, to establish the degree of correlation between citations and tweetations.

My primary tweetation metric was tw7 (cumulative number of tweetations 7 days after publication of the article, with day 0 being the publication date), a metric I also call *twimpact factor* or TWIF7 (see below).

The Pearson product moment correlation coefficients (*r*) for the raw citation versus tw7 tweetation counts were statistically significant on a 5% level for all journal issues, and ranged from .57 to .89 (Table 1). Pearson correlations between the logs of citations and logs of tweets, as well as Spearman rank correlation coefficients, were all statistically significant when articles across issues were combined, except for the rank correlation between Scopus citation counts and tweetations. When stratified by journal issue, the correlations for some issues were statistically significant for some computations, while for others they were not, perhaps due to a small sample size. Generally, the Google Scholar citations showed better correlations with tweetations than did Scopus citations (Table 1). The Spearman rank correlations (rank by citations versus rank by tw7) were statistically significant for only one issue, with rho = .51, *P* = .04 for issue 2/2010.

I also conducted analyses with other tweetation metrics (tw0, tw1, tw2, tw3, tw4, tw5, tw6, tw7, tw10, tw12, tw14, tw30, and tw365) and derived various metrics (tw365–tw7, ie, late-stage tweets; tw7–tw0, tw0/tw7 etc), which produced very similar correlation coefficients (data not shown).

**Table 1:** Correlation coefficients

		Issue (number of papers)									
		3/2009 (n = 19)		4/2009 (n = 11)		1/2010 (n = 8)		2/2010 (n = 17)		All (n = 55)	
		*r* or rho	*P* value	*r* or rho	*P* value	*r* or rho	*P* value	*r* or rho	*P* value	*r* or rho	*P* value
**Pearson correlation (** *r* **)**											
	CitGo-Tweets^a^	.57**	.01	.89***	<.001	.76*	.03	.68**	.003	.69***	<.001
	CitSc-Tweets^b^	.33	.17	.74**	.01	.65	.08	.51*	.04	.54***	<.001
	logCitGo-logTweets	.42	.08	.51	.11	.72*	.045	.49*	.048	.39**	.004
	logCitSc-logTweets	.03	.90	.41	.22	.53	.17	.47	.06	.31*	.02
**Spearman rank correlation (rho)**											
	CitGo-Tweets	.42	.07	.14	.68	.61	.11	.51*	.04	.36**	.006
	CitSc-Tweets	.06	.81	.11	.76	.44	.27	.42	.10	.22	.11

^a^ Citation count according to Google Scholar (CitGo) versus tweetation count (tw7).

^b^ Citation count according to Scopus (CitSc) versus tweetation count (tw7).

*P < .05, **P < .01, ***P < .001.

### Multivariate Analysis

In a linear regression model I tried to predict the log of the number of Google Scholar citations from the log of the number of tweets and time (days since publication of the first article in the sample of 55 articles). The regression equation was log(cit + 1) = 0.467 * log(tw7 + 1) + –.001 * days + 0.817, where cit is the number of citations, and tw7 is the cumulative number of tweetations at day 7. Both independent variables were significant predictors (*P* < .001), and the model explained 27% of the variation of citations (*R^2^* = .27). 

### Binary Analysis

Based on the observation that tweets were sent primarily during the early days after publication, I hypothesized that tw7, the cumulative number of tweetations by day 7 (perhaps as early as day 3), could be used as a diagnostic test to predict highly cited articles. *Highly tweeted* and *highly cited* are defined as articles in the 75^th^–100^th^ percentile of each journal issue; thus, the cut-off points on what constitutes highly tweeted or highly cited varied by issue (tweets: 11, 19, 34.8, 28.5; Google Scholar citations: 15, 9, 22.75, 15, for issues 3/2009, 4/2009, 1/2010, and 2/2010, respectively).

**Figure 10:**
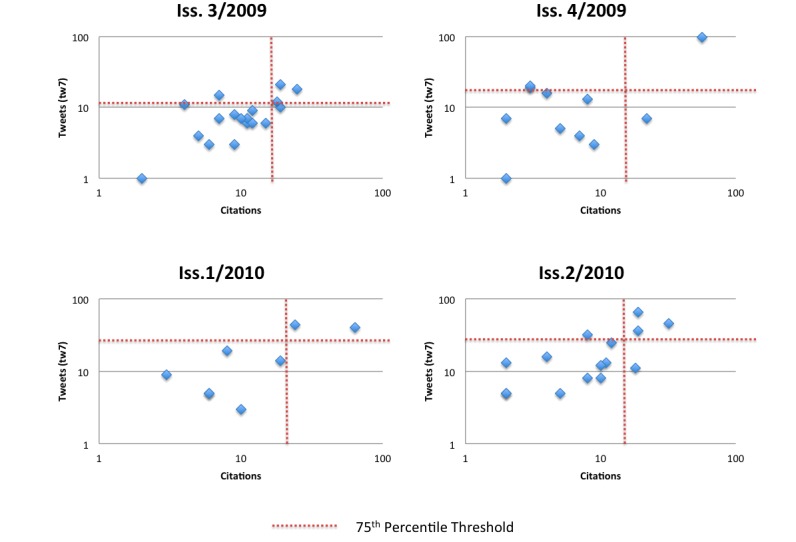
Correlations between citations in November 2011 (Google Scholar) and the cumulative number of early tweets by day 7 (tw7). Note the logarithmic scale. Articles with 0 tweets or 0 citations are not displayed here, because the log of 0 is not defined. However, conceptually they all fall into the lower left quadrant.

Table 2 is a 2 × 2 table categorizing articles into the four groups. Articles that were less frequently tweeted and not in the top-cited quartile are interpreted as true negatives (tn, lower left quadrant in Figure 10 and Table 2). Articles that were highly tweeted and highly cited are true positives (tp, upper right quadrant in Figure 10 and Table 2). Articles that were highly tweeted but not highly cited fall into the upper left quadrant and are referred to as false positives (fp). Finally, articles that were not highly tweeted but highly cited are false negatives (fn).

Using tweetation status (highly versus less tweeted) as a predictive test for citation status, this test identified 40 out of the 43 not highly cited articles, which translates to a 93% specificity (true-negative rate, tn/[tn + fp], 40/43). The test was able to correctly identify 9 out of the 12 highly cited papers, which corresponds to a 75% sensitivity (tp/[tp + fn], 9/12). Another way to express these results is to say that the positive predictive value (tp/[tp + fp]) or precision is 75%, meaning that if an article is highly tweeted (tests positive for social media impact), then there is a 75% likelihood that the article ends up in the top quartile of all articles of an issue, ranked by citations. The negative predictive value (tn/[tn + fn]) is 93% (40/43), meaning that if an article was not highly tweeted (tests negative for social media impact), then there is only a 7% (3/43) chance that it will fall into the top 25% of cited articles. Yet another way to express these results is to say that highly tweeted articles are almost 11 times more likely than less tweeted articles to be highly cited (9/12, 75% highly tweeted article are highly cited, while only 3/43, 7% of the less tweeted articles are highly cited; rate ratio 0.75/0.07 = 10.75, 95% confidence interval, 3.4–33.6).

There was a highly statistically significant association between citation status and tweetation status (Fisher exact test, *P* < .001).

I repeated this analysis for a range of different metrics such as tw*n* (cumulative number of tweetations after *n* days, with n = 0, 1–10, 12, 14, 30, or 365), and the number of late-response tweetations tw365–tw7. Starting on day 3 (tw3), the heuristic started to identify the same top-tweeted articles as tw7, indicating that the test is predictive as early as 3 days after publication. Choosing later days (letting tweetations accumulate for more than 7 days) or the late-response tweetations did not improve the test results (data not shown). 

**Table 2:** 2 × 2 table using top-tweeted articles as a predictor for top-cited articles

	Less cited (bottom 75%) n = 43	Highly cited (top 25%) n = 12
Highly tweeted (top 25%) n = 12	fp^a^ (n = 3) [Article ID 1223, 1163, 1281]	tp^b^ (n = 9) [Article ID 1252, 1303, 1270, 1249, 1337, 1376, 1371, 1350, 1549]
Less tweeted (bottom 75%) n = 43	tn^c^ (n = 40)	fn^d^ (n = 3) [Article ID 1086, 1256, 1357]

^a^ False positives.

^b^ True positives.

^c^ True negatives.

^d^ False negatives.

### Proposed Twitter-Based Metrics for Social Impact

The research reported here focuses on articles from one journal. However, I suggest that the metrics introduced here should be useful to measure the impact any article (or collections or sets of articles) has on Twitter, to gauge how much attention users pay to the topic of an article, to measure how the question and/or conclusions resonate with Twitter users, and ultimately to use them as proxies for social impact. Although I use Twitter as an example here, these metrics can be used in other social media (eg, Facebook status updates). The metrics presented here can also be generalized and applied to measure the impact of any issue (not just scholarly articles but, for example, current events and newspaper articles) on a social media user population.

#### Twimpact Factor (eg, tw7)

Using raw tweetation counts to compare the impact of different articles with each other is problematic, because the number of tweetations is a function of time since publication. Although the data suggest that after an initial period of 30 days tweetations usually occur only sporadically, the raw number of tweets should not be used when comparing articles with each other if they have been published on different dates. An average tweetation count per month since publication is possible to calculate (and is currently displayed on the JMIR Top Articles webpage, see Figure 1), but due to the highly skewed power law distribution, this average will always favor articles that have been published recently (within the last month).

I therefore propose to use (and have used in this paper) the twimpact factor tw*n* as a metric for immediate impact in social media, which is defined as the cumulative number of tweetations within *n* days after publication (eg, tw7 means total number of tweetations after n = 7 days). *Tweetations* can be replaced by *URL mentionings* if we apply this metric to other social media (URL being the URL or set of URLs of a specific article).

As a standard twimpact factor metric for an article on Twitter, I suggest (and JMIR will use in the future) tw7—that is, the absolute, cumulative number of tweetations an article receives by day *7* after publication (the day of publication is referred to as day 0). This is also a very practical metric: using a relatively short period of time makes the twimpact factor easier to compute, as the Twitter stream needs to be monitored for only 7 days.

I have shown that the number of new tweetations drops off rapidly after publication, even for the most highly cited papers. The immediate social media response is highly correlated with the later social media response; therefore, it is likely that the late response can be ignored. An even shorter period of time (3 days), tw3, was already sufficient in the sample to discriminate between highly cited and less cited articles, but I suggest a standard *n* of 7, which has the advantage that it always includes a weekend; thus, journal articles published toward the end of the week are less penalized for the weekend effect.

Any article, but also a collection of articles, can have a twimpact factor (eg, on a journal or issue level). JMIR is now monitoring the *c* *ollective t* *wimpact f* *actor* ctw*n*/*m* for each journal issue (where *n* is the number of days after publication tweetations accumulate, and *m* is the percentile), eg, ctw7/50 is the median (50^th^ percentile) of tw7 for all articles in the set. The ctw7/75 for JMIR issue 2/2010 is 29, meaning that the top 25% most-tweeted articles in issue 2/2010 were tweeted more than 29 times during the first week. We prefer to report the 75^th^ percentile instead of the mean or median (ctw7/50) because of the power distribution and because it seems a useful cut-off point to predict top-cited articles. At least in our sample, the practical meaning of the collective twimpact factor ctw7/75 is that articles with a tw7 greater than the ctw7/75 of a journal issue have a 75% likelihood of being top-cited (ending up in the top quartile of all articles of an issue, ranked by citations).

Note that the twimpact factor is an absolute measure counting tweetations; thus, just like for the journal impact factor, caveats apply. First, it is highly subject specific, so if comparisons are made between journals or even articles from the same journal, they should be made within a narrow subject category. An article on social media will more likely than an article about molecular biology be picked up by social media. Although within a specific field the twimpact factor may predict citations (predict which article is more likely to be highly cited), it would not be legitimate to compare the twimpact factor of an article on social media with a twimpact factor of an article about molecular biology, and conclude that the social media article will be more likely cited.

Second, similar to the caveat that journal impact factors should not be compared across different years, as the total number of citations is constantly growing, only articles that are published in a similar timeframe should be compared with each other (perhaps even 1 year is too long; thus, we made comparisons on a quarterly within-issue level). This is because both the number of Twitter users and the number of followers of a journal grow over time.

#### Tweeted Half-Life

The tweeted half-life (THL*n*) is defined as the point in time after publication by which half of all tweetations of that article within the first *n* days occur. As *n* I have used 30 days—that is, as the denominator I chose the total cumulative number of tweets within a 30-day period following the publication date. The THL*n* is the day when cumulatively half of these tweetations have occurred.

**Figure 11:**
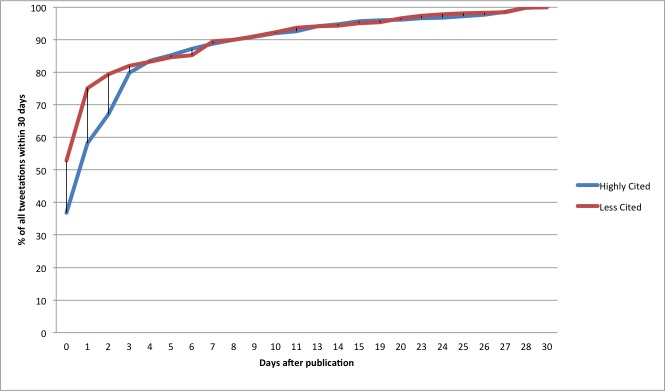
Tweetation curves: cumulative tweetations (tw*n*), as a proportion of all tweetations sent within 30 days.

In our sample, the THL*n* for the less-cited articles was 0 (53% of the tweets were tweeted on day 0), while the THL*n* of highly cited articles was 1 (on day 0, 37% of all tweetations occurred, while on day 1, 21% occurred, in total 58% by day 1). Figure 11 illustrates this. It may at first seem surprising that less-cited articles appear to show a quicker and proportionally higher response on the first days, but it should be kept in mind that the *absolute* counts of tweetations for more highly cited articles are higher than for the less-cited articles. Low-impact articles are tweeted and retweeted mainly on day 0 and day 1. Highly cited articles continue to be retweeted widely, which depresses the *relative* proportion of tweetations on days 0–3.

#### Twindex

As a final metric I propose (and JMIR will use) the twindex (tweetation index), which is a metric ranging from 0 to 100 indicating the relative standing of an article compared to other articles. I define the twindex_7_ of specific article as the rank percentile of this article when all articles (the specific article and articles from a comparator group) are ranked by the twimpact factor tw7. The comparator articles should be similar articles published in a similar time window (eg, other articles in the same issue, or the 19 articles published previously in the same journal). If an article has the highest twimpact factor tw7 among its comparator articles, it has a twindex of 100. If it has the lowest twimpact factor, it has a twindex of 0. In this study, articles with a twindex > 75 often also turned out to be the most-cited one.

## Discussion

### Principal Findings

To my knowledge, this is the first systematic, prospective, longitudinal article- and journal-level investigation of how mentionings (citations or tweetations) of scholarly articles in social media accumulate over time. It is also the first study correlating *altmetrics* to subsequent citations. I have discovered important regularities that will be very useful for others interested in applying and developing social media-based impact metrics, not only in the context of scientometrics.

This paper shows that buzz in the blogosphere is measurable, and that metrics can be derived that are somewhat correlated with citations. Citations from Google Scholar seem more closely correlated with tweetations than are citations from Scopus, which likely reflects the fact that Google Scholar includes a wider range of citing sources, especially from nonjournal documents [32]. The Spearman rank correlations are poorer than Pearson correlations, probably because among the less-tweeted articles tweetations are sparse, and often as few as 1 or 2 tweetations make a difference on the ranking of an article. The correlation is, however, strong enough that we can make surprisingly accurate binary predictions along the lines that highly tweeted articles are 11 times more likely to end up being highly cited.

Correlation is not causation, and it harder to decide whether extra citations are a *result* of the social media buzz, or whether it is the underlying quality of an article or newsworthiness that drives both the buzz and the citations—it is likely a combination of both. It is not inconceivable that exposure on Twitter leads to a few extra citations: social media are often used by scientists “to catch useful citations...scholars might not otherwise be exposed to” [24], and many scientists see the value of Twitter in being a constant live literature alert service crowdsourced from peers. Tweets contain hyperlinks to articles, and hyperlinks may affect the ranking in search engines such as Google and increase the visibility for researchers.

### Limitations of Twitter-Based Metrics

I suggest tweetations, twindex, and twimpact factor as metrics, which JMIR will publish and promote. These should be primarily seen as metrics for social impact (buzz, attentiveness, or popularity) and as a tool for researchers, journal editors, journalists, and the general public to filter and identify hot topics. Attentiveness to issues is a prerequisite for social change [33,34], and tweets are a useful metric to measure attentiveness to a specific scholarly publication. The data presented here also show that social impact is somewhat correlated with scientific impact, but there are important caveats. The correlation is far from perfect (as one can expect), and the *complementary* nature of the metrics needs to be stressed (as an aside, the complementary nature is also why the term altmetrics is not favored by this author—these metrics are probably not an *alternative*, but a *complement* to traditional citations). Popularity—which is one dimension of what tweet metrics are measuring—is an extremely useful (and revenue-predicting) measure for commercial enterprises such as the entertainment industry, but there are enormous pitfalls to applying metrics of popularity to health and science, if they are not qualified by or complemented with other metrics. While for funding organizations, journal editors, and research organizations it may be very valuable to know which topics resonate with the public (are popular and paid attention to), even though they did not receive a lot of citations (the articles in the false-positive group), there is a real danger that research topics or findings that are not trendy enough to resonate with the Twitter population—for example, research affecting disadvantaged populations that are not represented on Twitter—are marginalized. It is interesting that one of the false negatives (many citations, but few tweetations) included a paper dealing with a low-income elderly population [Article ID 1256]—exactly the population that is underrepresented on Twitter. On the other hand, publications that are “sexy,” trendy, or funny may receive huge exposure on Twitter, but may (or may not) have limited scientific value (a concept that is also not always accurately measured by citations).

Still, as mentioned earlier, there is enormous potential value for funding organizations, editors, and academic institutions to monitor these data, and to pay attention particularly to the false positives (high tweetations, low citations), as they may point to topics or questions that should perhaps be paid attention to. In our sample, the 3 articles that were highly tweeted but not highly cited (false positives; Article IDs 1223, 1163, 1281) all had a patient side to them, and consumers may have been the source of tweetations. Infoveillance of social media can be seen as a tool for public engagement in the discourse on what constitutes “important” research.

Finally, it must be acknowledged that there are journal-specific confounders at work that may limit the use of twimpact metrics, in particular if different journals are compared with each other (which is not currently done, but may be a future scenario). Journals cater to different communities and social networks, and when comparing how information propagates through online social networks, we may be measuring the structure of these networks and the attributes of these communities, rather than the attributes of the information itself. In other words, the number of tweetations is not a function of the intrinsic properties of the research article alone; rather, it is also influenced by factors related to the journal or venue it appears in, the community built around the journal, and how the scholarly information is marketed by the journal. But then again, the same is true for citations. 

### Limitations of This Study

While the results and metrics presented here are probably pivotal to paving the way to a new field of social media-based impact metrics, and while JMIR will increasingly use these approaches, the biggest question is whether our results and methods can be applied to other journals. JMIR is an ideal journal on which to experiment with altmetrics because it has a relatively high impact factor (ie, many traditional citation events) and—as a journal *about* the Internet and social media—it has a sophisticated readership that is generally ahead of the curve in adopting Web 2.0 tools. However, this also limits the generalizability of these results: what works for this journal may not work for other journals, in particular journals that are rarely cited (low impact factor) and that do not have an active Twitter user base. JMIR is a journal about information technology, and its readers may be more familiar with social media than readers of other journals are. Journals that publish non-Internet-related articles have probably far lower tweetation rates per article, and it is also less likely that people tweet about articles that are not open access. In fact, it has been argued that one key advantage of open access is that it facilitates knowledge dissemination among nonresearch users [35], and it is unlikely that articles from lower-impact subscription-based journals that are not accessible to a large number of users attract similar levels of tweetations. On the other hand, if tweetations about papers in subscription-based journals appear (eg, high-impact journals such as *Science* or *Nature*), it is likely that they were tweeted by expert users (scientist) who have access to the article; hence, they may be even more predictive for citations, because the general public is not (or to a lesser degree) part of the conversation.

The results presented here should be confirmed with tweets about other journals, as well as with future JMIR articles, and our group is currently conducting comparative analyses with other datasets. The hypothesis is that the results can be replicated for other journals as long as there is a large enough Twitter user base.

There are further, JMIR-specific caveats. First, as shown in Figure 1, JMIR ranks the top-tweeted articles on its website, and also sends out automatic tweets whenever a new article enters the top 10 in any of the monthly categories; both may have reinforced and amplified the response from Twitter users. Also, tweetations are a metric of the social media response; hence, the social media strategy of a journal likely has an impact on the results. Journals with an active social media presence and tweet alerts such as JMIR will have a higher uptake. JMIR followers have to click on only one button to retweet or modify these alerts (*seed tweets*). Journals that do not send out alerts for each article may have very different tweetation characteristics (eg, more late-stage tweetations). Further, the tweetation characteristics and rates are almost certainly influenced by the number of followers a journal has (JMIR currently has over 1000 followers) and, even more so, by lists and Twitter bots redistributing content to specific communities.

Researchers interested in using this new method and metric to compare different journals with each other should also be aware that the timing and frequency of article publication probably influence tweetation dynamics and rates (and may affect the strength of correlation between tweetations and citations). JMIR publishes articles as soon as they are ready, on different workdays of the week. As people tweet less during the weekend, the tweetation curve shown in Figure 2 may look slightly different for journals that always publish on Mondays (the drop-off may be less pronounced), compared with a journal that publishes always on a Friday (here, the drop-off may be more pronounced). However, the tw7 metric (cumulative tweetations over the course of a week) is probably robust enough to compare journals with different publication schedules. Seasonal effects are also evident. For example, issue 5/2010 (not shown and not included in our analysis) was a theme issue published shortly before Christmas, and in this issue all articles were published at once rather than spread out over multiple days; as a result, articles in this theme issue had very low tweetation rates.

The current report does not include a systematic qualitative analysis of tweet contents. However, a cursory scan through all the tweets suggests that the vast majority of tweets simply contained variants of the article title or the key conclusion, and rarely contained explicit positive sentiments (such as “Great article!”) or—even less common—negative sentiments (such as “questionable methods”—I have not seen any examples of the latter). This may be because the mere act of (re)tweeting an article is often an implicit endorsement or recommendation with which readers express their interest in and enthusiasm about a specific topic, support the research question and/or conclusion, or simply want to bring the article to the attention of their followers. Additional comments are not necessarily required to express this implicit endorsement. Also, with most tweets occurring on the day of publication, few readers will actually have had time to carefully read and appraise the entire paper beyond the title and perhaps abstract. While we originally thought of doing an automated sentiment analysis, the sparse nature of comments did not make this approach seem promising to elicit more specific data, although future studies using journals or articles with a high number of tweetations may want to take a close look at this question.

Future studies may also want to try to increase the specificity and sensitivity by focussing on specific types of twitter users, or taking into account the network structure and relative influence of the tweetation authors. JMIR publishes a tweets influence factor on its “Top Articles” Page (see Figure 1), which takes into account not only the number of tweets, but also the influence of the users who sent these tweets. The influence of users can be computed by the number of their followers and/or how often their tweets are retweeted, and more research is required to establish if these secondary metrics elicit additional information or are already reflected in the raw tweetation counts.

Another limitation is that the present analysis took into account first-order tweetations only. Tweets may contain links to blogs that in turn talk about articles, or may contain links to news articles that report on new research findings (second-order tweetations). According to Priem and Costello, about 50% are second-order tweetations [24]. This analysis did not capture these, as our tool strictly looks at tweetations with direct links to JMIR articles. We also did not capture links to other sites where JMIR articles may be hosted, including PubMed, PubMed Central, or DOI resolvers. Finally, twitter users commonly use URL shorteners, and while we retrieved some shortened URLs (by URL shorteners such as bit.ly), we may not have captured tweetations where the URL was shortened by less common shorteners. Thus, the true total number of tweetations was likely higher than what is reported here. On the other hand, there is no reason to believe that not counting these tweetations would introduce a bias.

In the current analysis each unique tweet was counted as 1 tweetation. Thus, multiple tweets sent by the same user about the same article would have been counted multiple times. This is not a problem in the current analysis, because multiple tweets with the same URL from the same user were quite rare. However, it is theoretically possible that—especially if tweetations become a more common method to rank and filter articles—authors may start to “game” the system by sending multiple tweets about their own article to create more exposure for their articles. Thus, for any use case with serious implications for authors (eg, if tweetations become a more accepted and common early metrics for social impact), a tweetation should be defined as an URL mentioned by a distinct unique user.

### Conclusions

It is a fascinating and compelling finding that the collective intelligence of Twitter users can, within limitations, predict citations, which normally take years to accumulate.

**Figure 12:**
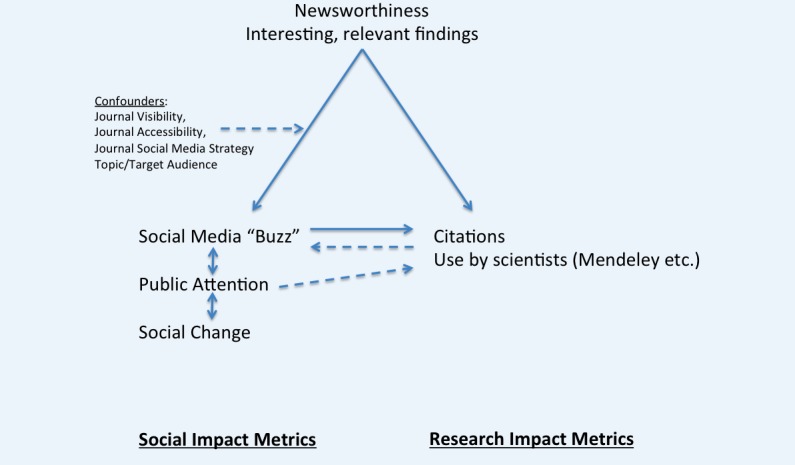
Model of the relationship between social impact and research impact metrics.

It should be stressed again that one should neither expect nor hope for perfect correlation. Tweetations should be primarily seen as a metric for social impact and knowledge translation (how quickly new knowledge is taken up by the public) as well as a metric to measure public interest in a specific topic (what the public is paying attention to), while citations are primarily a metric for scholarly impact. Both are somewhat *correlated*, as shown here, but tweetations and citations measure different concepts, and measure uptake by or interest of different audiences (Figure 12). The correlation and mutual interaction between these audiences is illustrated in Figure 12 with bidirectional arrows, which point from “social media buzz” to “citations” (scientists being influenced by social media buzz), and from “use by scientists” to “social media buzz” (scientists creating buzz on Twitter), illustrating the mutual influence of these audiences and metrics.

So if not (primarily) as a proxy or early indicators for citations, how should or could tweetations be used? What are the use cases?

First, social media metrics can be easily used by scholars, institutions, and journals to monitor the overall impact of research in a timely manner, keeping in mind the caveats and limitations listed above. Second, these metrics could be used to evaluate different methods of knowledge dissemination. One could design studies where different methods of promoting an article (or other URLs, for example public health intervention websites) are evaluated, with the twimpact factor as an outcome measure. Third, social media impact metrics can also be used as a filter to direct users to research articles that the public or research communities are paying attention to. A website displaying real-time social impact metrics such as twimpact factors of current research articles may be useful for a wide range of potential audiences, including journalists, journal editors, researchers, public health officials, and patients, to direct them to topics and research that resonate with the public.

### A Standing Call for Papers

More research is required to assess the robustness of these social media metrics and their ability to detect signals among the noise of social media chatter, for scientometric purposes or other use cases in infodemiologic research. As mentioned earlier, the metrics and regularities presented here not only have applications for scientometrics, but also may be used to measure the dynamics and “half-life” of other issues or events discussed on Twitter or social media in general.

To stimulate and encourage innovation, research and development in this area, JMIR hereby issues a standing call for papers, welcoming empirical and viewpoint papers on the broad topic of infometrics or infodemiology metrics (or altmetrics, in the context of scientometrics), in particular with concrete use cases and data from health-related fields or journals. We look forward to publishing more research on what we feel are important methodological foundations for exploiting crowdsourcing and collective intelligence themes within the field of Internet research and science 2.0.

## Multimedia Appendix 1

Dataset of 4208 tweets mentioning JMIR articles between July 24, 2008 and November 20, 2011. Real-time, daily updated summary article-level metrics for all JMIR articles including citation and tweetation counts, twimpact factors, twindex etc. can be downloaded from http://www.jmir.org/stats/articles?format=csv. Researchers interested in using and further analyzing these data are encouraged to contact the author.

## Multimedia Appendix 2

Included references.

## Abbreviations

API: application programming interface
ctw7/75: collective twimpact factor for the 75th percentile of cumulative tweets within first 7 days of publication for all articles in a set of articles
DOI: digital object identifier
fn: false negative
fp: false positive
JMIR: Journal of Medical Internet Research
PLoS: Public Library of Science
THLn: tweeted half-life
tn: true negative
tp: true positive
tw*n*: cumulative number of tweets within *n* days of publication of an article, with day 0 being the publication date; tw7 is also called the twimpact factor

## References

[1] Hirsch JE (2005). An index to quantify an individual's scientific research output. Proc Natl Acad Sci U S A.

[2] Garfield E (2006). The history and meaning of the journal impact factor. JAMA.

[3] Rossner M, Van Epps H, Hill E (2007). Show me the data. J Cell Biol.

[4] PLoS Editors (2006). The impact factor game. It is time to find a better way to assess the scientific literature. PLoS Med.

[5] Smith R (2001). Measuring the social impact of research. BMJ.

[6] Niederkrotenthaler T, Dorner TE, Maier M (2011). Development of a practical tool to measure the impact of publications on the society based on focus group discussions with scientists. BMC Public Health.

[7] Thelwall M (2009). Introduction to Webometrics: Quantitative Web Research for the Social Sciences. Synthesis Lectures on Information Concepts, Retrieval, and Services.

[8] Vaughan, L (2005). Web link counts correlate with ISI impact factors: Evidence from two disciplines. Proceedings of the American Society for Information Science and Technology.

[9] Vaughan L, Shaw D (2003). Bibliographic and Web citations: What is the difference?. Journal of the American Society for Information Science and Technology.

[10] Vaughan L, Shaw D (2005). Web citation data for impact assessment: A comparison of four science disciplines. Journal of the American Society for Information Science and Technology.

[11] Kousha K, Thelwall M, Rezaie S (2010). Using the Web for research evaluation: The Integrated Online Impact indicator. Journal of Informetrics.

[12] Thelwall M, Kousha K (2008). Online presentations as a source of scientific impact?: An analysis of PowerPoint files citing academic journals. Journal of the American Society for Information Science and Technology.

[13] Kousha K, Thelwall M (2009). Google Book Search: Citation analysis for social science and the humanities. Journal of the American Society for Information Science and Technology.

[14] Priem J, Hemminger BM (2010). Scientometrics 2.0: Toward new metrics of scholarly impact on the social Web. First Monday.

[15] Li X, Thelwall M, Giustini D (2011). Validating Online Reference Managers for Scholarly Impact Measurement (FP).

[16] Priem J, Taraborelli D, Groth P, Neylon C altmetrics.org.

[17] Eysenbach G (2011). Infodemiology and infoveillance tracking online health information and cyberbehavior for public health. Am J Prev Med.

[18] Eysenbach G (2009). Infodemiology and infoveillance: framework for an emerging set of public health informatics methods to analyze search, communication and publication behavior on the Internet. J Med Internet Res.

[19] Hubbard DW (2011). Pulse: The New Science of Harnessing Internet Buzz to Track Threats and Opportunities.

[20] Eysenbach G (2006). Infodemiology: tracking flu-related searches on the web for syndromic surveillance. AMIA Annu Symp Proc.

[21] Ginsberg J, Mohebbi MH, Patel RS, Brammer L, Smolinski MS, Brilliant L (2009). Detecting influenza epidemics using search engine query data. Nature.

[22] Chew C, Eysenbach G (2010). Pandemics in the age of Twitter: content analysis of Tweets during the 2009 H1N1 outbreak. PLoS One.

[23] Asur S, Huberman BA Predicting the future with social media.

[24] Priem J, Costello KL (2010). How and why scholars cite on Twitter. Proceedings of the American Society for Information Science and Technology.

[25] Letierce J, Passant A, Decker S, Breslin JG (2010). Understanding how Twitter is used to spread scientific messages. http://journal.webscience.org/314/2/websci10_submission_79.pdf.

[26] Weller K, Dröge E, Puschmann C (2011). Citation analysis in Twitter: Approaches for defining and measuring information flows within tweets during scientific conferences.

[27] Ross C, Terras M, Warwick C, Welsh A (2011). UCL, self-archived draft version.

[28] Weller K, Puschmann C (2011). Twitter for scientific communication: How can citations/references be identified and measured?. Proceedings of the Poster Session.

[29] Priem J, Costello K, Dzuba T (2011). Prevalence and use of Twitter among scholars. self-archived Poster.

[30] Priem J, Piwowar H, Hemminger B (2011). Altmetrics in the wild: An exploratory study of impact metrics based on social media.

[31] Newmann MEJ (2005). Power laws, Pareto distributions and Zipf's law. Contemporary Physics.

[32] Kousha K, Thelwall M (2007). Sources of Google Scholar citations outside the Science Citation Index: A comparison between four science disciplines. Scientometrics.

[33] Ripberger JT (2010). Social Science Research Network.

[34] Ripberger JT (2011). Capturing Curiosity: Using Internet Search Trends to Measure Public Attentiveness. Policy Studies Journal.

[35] Eysenbach G (2006). The open access advantage. J Med Internet Res.

