# Correction: Can Tweets Predict Citations? Metrics of Social Impact Based on Twitter and Correlation with Traditional Metrics of Scientific Impact

**DOI:** 10.2196/jmir.2041

**Published:** 2012-01-04

**Authors:** Gunther Eysenbach

**Affiliations:** ^1^University Health NetworkCentre for Global eHealth Innovation & Techna InstituteToronto, ONCanada; ^2^Institute for Health Policy, Management, and EvaluationUniversity of TorontoToronto, ONCanada; ^3^JMIR Publications Inc.Toronto, ONCanada

A minor error in the references section in the originally published version of the editorial by Eysenbach (J Med Internet Res 2011;13[4]:e123) on the relationship between citations and tweetations has been corrected; in addition, references being part of the dataset are no longer cited as “references”. The now corrected problem with the references was a “formatting/presentation” problem only and had no impact on the study findings. The originally published article stated correctly that all 55 articles published between issue 3/2009 and 2/2010 were included, but the cited references erroneously contained 12 additional references from issue 2/2009, which were not part of the analysis, for the reasons described in the article (sparse tweetations pre-issue 3/2009). In the corrected version we have not only removed these extra 12 references (31-42), but we also took the opportunity to move all other references of included articles (43-97) into a new Multimedia Appendix 2, no longer citing them in the “References” section. We now refer to them in the paper by article ID (last 4-digits of the DOI), where we previously used in-text citations (Table 2 and Discussion). While there was nothing wrong with the way the articles were cited previously, and while we think that citing the JMIR articles whose impact we discuss in the paper is proper and necessary, we want to avoid any potential impression that this editorial artificially skews JMIR's future impact factor. One way to avoid this is to move the references to a separate file. The original decision to cite them as references was made for the sake of convenience for our readers, to prevent them from having to look up the references in a separate file or by DOI. JMIR has no space limitations and generally prefers to cite references in the article rather than in an Appendix; for readers downloading a PDF file it is more convenient to have all references in a single file rather than having to download a separate Appendix. The decision to now move these references into a Multimedia Appendix was made after a reader and publishing colleague pointed out that citing these articles may increase JMIR’s impact factor. Although none of the two peer-reviewers, both experts in scientometrics, were originally concerned about citing the included articles as references, and even though any potential additional impact factor points after the decimal point caused by the original editorial would probably have been neglible (after all, these articles are already highly cited: altogether, 638 times, according to Google Scholar), and even though Thomson Reuters also publishes a journal impact factor that excludes journal self-citations, we wish to avoid any potential debate or uncertainty on what proportion of future JMIR impact factors were caused by this editorial, and have therefore decided to pre-emptively move these references into a separate file (Multimedia Appendix 2). The article correction was made on January 4, 2012, before submission to PubMed Central, Swets and other content aggregators and databases, and before indexing by Thomson Reuters. Having to remove references from a manuscript to preserve the validity of a journal-level impact metric is somewhat troubling, but if anything, then this perhaps illustrates the limitations and tyranny of the impact factor, and why we should consider additional metrics.

