# Supplementary material for: Can Tweets Predict Citations? Metrics of Social Impact Based on Twitter and Correlation with Traditional Metrics of Scientific Impact
Source: J Med Internet Res. 2011 Dec 16;13(4):e123. doi: 10.2196/jmir.2012 (PMC3278109; doi:10.2196/jmir.2012)
Supplement: Supplementary file 2 [file jmir_v13i4e123_app2.pdf]

Multimedia Appendix 2: Included references (JMIR issues 3/2009-2/2010)

Asterisks next to article IDs denote that the article is top-cited (see also Figure 8): ** top 25th citation percentile within issue by both Scopus and Google Scholar citation counts * top 25th citation percentile according to Google Scholar only, (*) top 25th citation percentile according to Scopus only.

Article ID (last 4 digits of DOI)	Article
1123	Himmel W, Reincke U, Michelmann HW. Text mining and natural language processing approaches for automatic categorization of lay requests to web-based expert forums. J Med Internet Res 2009;11(3):e25 [FREE Full text] [CrossRef] [Medline]
1142(*)	Takahashi Y, Uchida C, Miyaki K, Sakai M, Shimbo T, Nakayama T. Potential benefits and harms of a peer support social network service on the internet for people with depressive tendencies: qualitative content analysis and social network analysis. J Med Internet Res 2009;11(3):e29 [FREE Full text] [CrossRef] [Medline]
1134(*)	Donker T, van Straten A, Marks I, Cuijpers P. A brief Web-based screening questionnaire for common mental disorders: development and validation. J Med Internet Res 2009;11(3):e19 [FREE Full text] [CrossRef] [Medline]
1084	Vandelanotte C, Sugiyama T, Gardiner P, Owen N. Associations of leisure-time internet and computer use with overweight and obesity, physical activity and sedentary behaviors: cross-sectional study. J Med Internet Res 2009;11(3):e28 [FREE Full text] [CrossRef] [Medline]
1179	Wanner M, Martin-Diener E, Braun-Fahrländer C, Bauer G, Martin BW. Effectiveness of active-online, an individually tailored physical activity intervention, in a real-life setting: randomized controlled trial. J Med Internet Res 2009;11(3):e23 [FREE Full text] [CrossRef] [Medline]
1139	Slootmaker SM, Chinapaw MJ, Schuit AJ, Seidell JC, Van Mechelen W. Feasibility and effectiveness of online physical activity advice based on a personal activity monitor: randomized controlled trial. J Med Internet Res 2009;11(3):e27 [FREE Full text] [CrossRef] [Medline]
1183	Bot M, Milder IE, Bemelmans WJ. Nationwide implementation of Hello World: a Dutch email-based health promotion program for pregnant women. J Med Internet Res 2009;11(3):e24 [FREE Full text] [CrossRef] [Medline]
1222	Waters EA, Sullivan HW, Nelson W, Hesse BW. What is my cancer risk? How internet-based cancer risk assessment tools communicate individualized risk estimates to the public: content analysis. J Med Internet Res 2009;11(3):e33 [FREE Full text] [CrossRef] [Medline]
1221	Andersen P, Lindgaard AM, Prgomet M, Creswick N, Westbrook JI. Mobile and fixed computer use by doctors and nurses on hospital wards: multi-method study on the relationships between clinician role, clinical task, and device choice. J Med Internet Res 2009;11(3):e32 [FREE Full text] [CrossRef] [Medline]
1164	Simon SR, Evans JS, Benjamin A, Delano D, Bates DW. Patients' attitudes toward electronic health information exchange: qualitative study. J Med Internet Res 2009;11(3):e30 [FREE Full text] [CrossRef] [Medline]
1166	Wright A, McGlinchey EA, Poon EG, Jenter CA, Bates DW, Simon SR. Ability to generate patient registries among practices with and without electronic health records. J Med Internet Res 2009;11(3):e31 [FREE Full text] [CrossRef] [Medline]

1248	Brigham J, Lessov-Schlaggar CN, Javitz HS, Krasnow RE, McElroy M, Swan GE. Test-retest reliability of web-based retrospective self-report of tobacco exposure and risk. J Med Internet Res 2009;11(3):e35 [FREE Full text] [CrossRef] [Medline]
1252**	Scherr D, Kastner P, Kollmann A, Hallas A, Auer J, Krappinger H, MOBITEL Investigators. Effect of home-based telemonitoring using mobile phone technology on the outcome of heart failure patients after an episode of acute decompensation: randomized controlled trial. J Med Internet Res 2009;11(3):e34 [FREE Full text] [CrossRef] [Medline]
1097	Harris PR, Sillence E, Briggs P. The effect of credibility-related design cues on responses to a web-based message about the breast cancer risks from alcohol: randomized controlled trial. J Med Internet Res 2009;11(3):e37 [FREE Full text] [CrossRef] [Medline]
1086**	Stopponi MA, Alexander GL, McClure JB, Carroll NM, Divine GW, Calvi JH, et al. Recruitment to a randomized web-based nutritional intervention trial: characteristics of participants compared to non-participants. J Med Internet Res 2009;11(3):e38 [FREE Full text] [CrossRef] [Medline]
1223	Hill-Kayser CE, Vachani C, Hampshire MK, Jacobs LA, Metz JM. An internet tool for creation of cancer survivorship care plans for survivors and health care providers: design, implementation, use and user satisfaction. J Med Internet Res 2009;11(3):e39 [FREE Full text] [CrossRef] [Medline]
1193	Agha Z, Roter DL, Schapira RM. An evaluation of patient-physician communication style during telemedicine consultations. J Med Internet Res 2009;11(3):e36 [FREE Full text] [CrossRef] [Medline]
1303*	Griffiths KM, Calear AL, Banfield M, Tam A. Systematic review on Internet Support Groups (ISGs) and depression (2): What is known about depression ISGs? J Med Internet Res 2009;11(3):e41 [FREE Full text] [CrossRef] [Medline]
1270*	Griffiths KM, Calear AL, Banfield M. Systematic review on Internet Support Groups (ISGs) and depression (1): Do ISGs reduce depressive symptoms? J Med Internet Res 2009;11(3):e40 [FREE Full text] [CrossRef] [Medline]
1294	Costin DL, Mackinnon AJ, Griffiths KM, Batterham PJ, Bennett AJ, Bennett K, et al. Health e-cards as a means of encouraging help seeking for depression among young adults: randomized controlled trial. J Med Internet Res 2009;11(4):e42 [FREE Full text] [CrossRef] [Medline]
1263	Sacks N, Cabral H, Kazis LE, Jarrett KM, Vetter D, Richmond R, et al. A web-based nutrition program reduces health care costs in employees with cardiac risk factors: before and after cost analysis. J Med Internet Res 2009;11(4):e43 [FREE Full text] [CrossRef] [Medline]
1256**	Kim EH, Stolyar A, Lober WB, Herbaugh AL, Shinstrom SE, Zierler BK, et al. Challenges to using an electronic personal health record by a low-income elderly population. J Med Internet Res 2009;11(4):e44 [FREE Full text] [CrossRef] [Medline]
1244	Joshi A, Arora M, Dai L, Price K, Vizer L, Sears A. Usability of a patient education and motivation tool using heuristic evaluation. J Med Internet Res 2009;11(4):e47 [FREE Full text] [CrossRef] [Medline]
1225	Jones RB, Maramba I, Boulos MN, Alexander T. Use of live interactive webcasting for an international postgraduate module in ehealth: case study evaluation. J Med Internet Res 2009;11(4):e46 [FREE Full text] [CrossRef] [Medline]
1220	Pak R, Price MM, Thatcher J. Age-sensitive design of online health information: comparative usability study. J Med Internet Res 2009;11(4):e45 [FREE Full text] [CrossRef] [Medline]

1249**	Chou WY, Hunt YM, Beckjord EB, Moser RP, Hesse BW. Social media use in the United States: implications for health communication. J Med Internet Res 2009;11(4):e48 [FREE Full text] [CrossRef] [Medline]
1163	Eddens KS, Kreuter MW, Morgan JC, Beatty KE, Jasim SA, Garibay L, et al. Disparities by race and ethnicity in cancer survivor stories available on the web. J Med Internet Res 2009;11(4):e50 [FREE Full text] [CrossRef] [Medline]
1311	Gracia E, Herrero J. Internet use and self-rated health among older people: a national survey. J Med Internet Res 2009;11(4):e49 [FREE Full text] [CrossRef] [Medline]
1258	Peiris DP, Joshi R, Webster RJ, Groenestein P, Usherwood TP, Heeley E, et al. An electronic clinical decision support tool to assist primary care providers in cardiovascular disease risk management: development and mixed methods evaluation. J Med Internet Res 2009;11(4):e51 [FREE Full text] [CrossRef] [Medline]
1278	Tian H, Brimmer DJ, Lin JM, Tumpey AJ, Reeves WC. Web usage data as a means of evaluating public health messaging and outreach. J Med Internet Res 2009;11(4):e52 [FREE Full text] [CrossRef] [Medline]
1337**	Wiecha J, Heyden R, Sternthal E, Merialdi M. Learning in a virtual world: experience with using second life for medical education. J Med Internet Res 2010;12(1):e1 [FREE Full text] [CrossRef] [Medline]
1298	Bexelius C, Löf M, Sandin S, Trolle Lagerros Y, Forsum E, Litton JE. Measures of physical activity using cell phones: validation using criterion methods. J Med Internet Res 2010;12(1):e2 [FREE Full text] [CrossRef] [Medline]
1361	Wanner M, Martin-Diener E, Bauer G, Braun-Fahrländer C, Martin BW. Comparison of trial participants and open access users of a web-based physical activity intervention regarding adherence, attrition, and repeated participation. J Med Internet Res 2010;12(1):e3 [FREE Full text] [CrossRef] [Medline]
1376**	Webb TL, Joseph J, Yardley L, Michie S. Using the internet to promote health behavior change: a systematic review and meta-analysis of the impact of theoretical basis, use of behavior change techniques, and mode of delivery on efficacy. J Med Internet Res 2010;12(1):e4 [FREE Full text] [CrossRef] [Medline]
1284	Kazemi A, Fors UG, Tofighi S, Tessma M, Ellenius J. Physician order entry or nurse order entry? Comparison of two implementation strategies for a computerized order entry system aimed at reducing dosing medication errors. J Med Internet Res 2010;12(1):e5 [FREE Full text] [CrossRef] [Medline]
1215	Overberg R, Otten W, de Man A, Toussaint P, Westenbrink J, Zwetsloot-Schonk B. How breast cancer patients want to search for and retrieve information from stories of other patients on the internet: an online randomized controlled experiment. J Med Internet Res 2010;12(1):e7 [FREE Full text] [CrossRef] [Medline]
1149	Bessière K, Pressman S, Kiesler S, Kraut R. Effects of internet use on health and depression: a longitudinal study. J Med Internet Res 2010;12(1):e6 [FREE Full text] [CrossRef] [Medline]
1347	Binks M, van Mierlo T. Utilization patterns and user characteristics of an ad libitum Internet weight loss program. J Med Internet Res 2010;12(1):e9 [FREE Full text] [CrossRef] [Medline]
1191	Damman OC, van den Hengel YK, van Loon AJ, Rademakers J. An international comparison of web-based reporting about health care quality: content analysis. J Med Internet Res 2010;12(2):e8 [FREE Full text]

	[CrossRef] [Medline]
1371**	Morris ME, Kathawala Q, Leen TK, Gorenstein EE, Guilak F, Labhard M, et al. Mobile therapy: case study evaluations of a cell phone application for emotional self-awareness. J Med Internet Res 2010;12(2):e10 [FREE Full text] [CrossRef] [Medline]
1520	Ivanitskaya L, Brookins-Fisher J, O Boyle I, Vibbert D, Erofeev D, Fulton L. Dirt cheap and without prescription: how susceptible are young US consumers to purchasing drugs from rogue internet pharmacies? J Med Internet Res 2010;12(2):e11 [FREE Full text] [CrossRef] [Medline]
1260	Ahmad F, Skinner HA, Stewart DE, Levinson W. Perspectives of family physicians on computer-assisted health-risk assessments. J Med Internet Res 2010;12(2):e12 [FREE Full text] [CrossRef] [Medline]
1251	Wilson PM, Petticrew M, Calnan M, Nazareth I. Effects of a financial incentive on health researchers' response to an online survey: a randomized controlled trial. J Med Internet Res 2010;12(2):e13 [FREE Full text] [CrossRef] [Medline]
1356	Weitzman ER, Kaci L, Mandl KD. Sharing medical data for health research: the early personal health record experience. J Med Internet Res 2010;12(2):e14 [FREE Full text] [CrossRef] [Medline]
1307	Joseph-Williams N, Evans R, Edwards A, Newcombe RG, Wright P, Grol R, et al. Supporting informed decision making online in 20 minutes: an observational web-log study of a PSA test decision aid. J Med Internet Res 2010;12(2):e15 [FREE Full text] [CrossRef] [Medline]
1314	Ip EJ, Barnett MJ, Tenerowicz MJ, Perry PJ. The touro 12-step: a systematic guide to optimizing survey research with online discussion boards. J Med Internet Res 2010;12(2):e16 [FREE Full text] [CrossRef] [Medline]
1267	Nordfeldt S, Hanberger L, Berterö C. Patient and parent views on a Web 2.0 Diabetes Portal--the management tool, the generator, and the gatekeeper: qualitative study. J Med Internet Res 2010;12(2):e17 [FREE Full text] [CrossRef] [Medline]
1350**	Van De Belt TH, Engelen LJ, Berben SA, Schoonhoven L. Definition of Health 2.0 and Medicine 2.0: a systematic review. J Med Internet Res 2010;12(2):e18 [FREE Full text] [CrossRef] [Medline]
1549**	Wicks P, Massagli M, Frost J, Brownstein C, Okun S, Vaughan T, et al. Sharing health data for better outcomes on PatientsLikeMe. J Med Internet Res 2010;12(2):e19 [FREE Full text] [CrossRef] [Medline]
1281	Santana S, Lausen B, Bujnowska-Fedak M, Chronaki C, Kummervold PE, Rasmussen J, et al. Online communication between doctors and patients in Europe: status and perspectives. J Med Internet Res 2010;12(2):e20 [FREE Full text] [CrossRef] [Medline]
1357**	Paré G, Moqadem K, Pineau G, St-Hilaire C. Clinical effects of home telemonitoring in the context of diabetes, asthma, heart failure and hypertension: a systematic review. J Med Internet Res 2010;12(2):e21 [FREE Full text] [CrossRef] [Medline]
1390	Gentles SJ, Lokker C, McKibbon KA. Health information technology to facilitate communication involving health care providers, caregivers, and pediatric patients: a scoping review. J Med Internet Res 2010;12(2):e22 [FREE Full text] [CrossRef] [Medline]
1286	Samoocha D, Bruinvels DJ, Elbers NA, Anema JR, van der Beek AJ. Effectiveness of web-based interventions on patient empowerment: a systematic review and meta-analysis. J Med Internet Res 2010;12(2):e23 [FREE Full text] [CrossRef] [Medline]
1409	Enwald HP, Huotari ML. Preventing the obesity epidemic by second

	generation tailored health communication: an interdisciplinary review. J Med Internet Res 2010;12(2):e24 [FREE Full text] [CrossRef] [Medline]
1396	Lau AY, Coiera E, Zrimec T, Compton P. Clinician search behaviors may be influenced by search engine design. J Med Internet Res 2010;12(2):e25 [FREE Full text] [CrossRef] [Medline]
